# Comparison of the effectiveness of three different conservative methods in myogenous temporomandibular disorders

**DOI:** 10.1590/1806-9282.20242102

**Published:** 2025-07-07

**Authors:** Nazım Tolgahan Yıldız, Zafer Erden, Gürsoy Coşkun, Filiz Can, Hakan Hıfzı Tüz

**Affiliations:** 1 Karamanoglu Mehmetbey University, Faculty of Health Sciences, Department of Physiotherapy and Rehabilitation – Karaman, Turkey.; 2 Hacettepe University, Faculty of Physical Therapy and Rehabilitation – Ankara, Turkey.; 3 Hacettepe University, Faculty of Dentistry, Department of Oral and Maxillofacial Surgery – Ankara, Turkey.

**Keywords:** Conservative treatments, Masticatory muscles, Surface electromyography, Temporomandibular disorders

## Abstract

**OBJECTIVE:**

The aim of this study was to compare the effectiveness of exercise, transcutaneous electrical nerve stimulation, and manual therapy on pain intensity, maximum mouth opening, and masticatory muscle activity-during chewing of the masseter and temporalis anterior in myogenous temporomandibular disorders.

**METHODS:**

A total of 51 myogenous temporomandibular disorders patients were randomly assigned to three groups: exercise group, transcutaneous electrical nerve stimulation group, and manual therapy group. For 6 weeks, exercise group received exercise only, transcutaneous electrical nerve stimulation group received exercise+transcutaneous electrical nerve stimulation, and manual therapy group received exercise+manual therapy. At baseline and after 6-week treatment, pain intensity was assessed using a visual analog scale, maximum mouth opening using a millimeter ruler, and masticatory muscle activity-during chewing of the masseter and temporalis anterior using surface electromyography.

**RESULTS:**

After treatment, significant improvements in pain intensity, maximum mouth opening, and masticatory muscle activity-during chewing values were found in all the three groups. The greatest improvements were seen in manual therapy group. The decreases in pain intensity and increases in masticatory muscle activity-during chewing values were statistically significantly higher in transcutaneous electrical nerve stimulation group than in exercise group (p<0.05). The increases in maximum mouth opening were similar in transcutaneous electrical nerve stimulation group and exercise group.

**CONCLUSION:**

Exercise is an effective method for improving pain intensity, maximum mouth opening, and masticatory muscle activity-during chewing in myogenous temporomandibular disorders. Combining exercise with manual therapy may provide the highest therapeutic effect on these parameters. Combining exercise with transcutaneous electrical nerve stimulation may also lead to further improvements in pain intensity and masticatory muscle activity-during chewing.

## INTRODUCTION

Temporomandibular disorders (TMDs) are defined as a group of musculoskeletal disorders affecting the temporomandibular joint (TMJ), masticatory muscles, and associated structures^1,2^. The main signs and symptoms of TMD are limited mouth opening, pain, difficulty chewing, and headaches. Patients’ most common complaints are pain and restricted movements of the jaw^2,3^. The prevalence of TMDs, which is most common in individuals between the ages of 18 and 45 years and more common in women, can be as high as 25%^3,4^. Myogenous TMD (mTMD) is the most prevalent subdiagnostic group of TMDs, characterized by severe pain and functional limitations^3,5^. TMDs significantly impact individuals’ health and quality of life by causing functional limitations in the orofacial system^1,3,4^. Therefore, the effective management of TMDs is critical^3,5,6^.

The current literature recommends the primary use of conservative approaches such as patient education, therapeutic exercises, occlusal splints, manual therapy (MT), and electrotherapy modalities such as transcutaneous electrical nerve stimulation (TENS) to improve clinical symptoms in the treatment of TMDs. It has been emphasized that these approaches, which have a low risk of side effects, are effective in improving pain intensity, maximum mouth opening (MMO), and masticatory muscle activity (MMA) in TMDs. However, it remains unclear which of the conservative approaches, such as exercise, TENS, and MT, is more effective for improving pain intensity, MMO, and MMA. In previous studies, controversial findings have been reported in this regard, and the literature has recommended further studies comparing the efficacy of conservative methods such as exercise, TENS, and MT in the improvement of pain intensity and MMO, especially in mTMD^5,7-9^.

TMD pain reduces the MMA to protect the masticatory system from trauma, which can reduce muscle activation and strength, resulting in chewing difficulties. By effectively treating TMD pain, MMA can be increased, improving the masticatory function of patients^3^. The MMA-during chewing (MMA-DC) can be objectively analyzed using surface electromyography (sEMG) to assess the effectiveness of the treatment on the MMA in TMDs^3,5^. Determining the most effective conservative method for improving MMA-DC may provide valuable insights into providing more effective treatment for patients and establishing assessment and treatment programs, particularly in mTMD^3,5^. However, to the authors’ best knowledge, no comparison has been previously made on determining the most effective intervention among exercise, TENS, and MT for improving the sEMG-assessed MMA-DC of masticatory muscles in patients with mTMD^5,7^. Clinical trials on this topic are very rare and have only examined the short-term or immediate effects of a single conservative treatment on MMA in mTMD^5,10-14^. Given all this, this study aimed to compare the effectiveness of exercise, TENS, and MT on pain intensity, MMO, and MMA-DC of the masseter and temporalis anterior (TA) muscles in mTMD patients.

## METHODS

### Study design

This randomized controlled study was conducted at the Faculty of Physical Therapy and Rehabilitation, Hacettepe University. The study protocol was approved by the Ankara City Hospital No.1 Research Ethics Committee (number: E1/907/2020). Participants provided written informed consent for the study, which followed the Consort Statement guidelines and the Declaration of Helsinki.

### Participants

The study enrolled patients diagnosed with mTMD using the Diagnostic Criteria for TMDs, who underwent clinical and radiological examinations by a specialist dentist experienced in TMDs at the Faculty of Dentistry, Hacettepe University. The study included patients who were aged 18–50 years with unilateral mTMD, had complaints for over 6 months, had full dentition, and were not receiving TMD treatment in the last 3 months. Patients with a history of trauma, surgery, toothache, psychiatric or systemic illness, medication use, pacemaker use, or pregnancy were excluded.

### Interventions

The participants were randomly divided into three groups: exercise group (EG), TENS group (TG), and MT group (MTG). EG received exercise therapy alone for 6 weeks, while TG received TENS therapy for 30 min each session, 3 days a week, for 6 weeks besides exercise therapy. MTG received MT for 30 min each session, 3 days a week, for 6 weeks besides exercise therapy. Before performing the exercises, the participants underwent TENS therapy in TG and MT techniques in MTG. A specialist physiotherapist was responsible for implementing and controlling all treatments. Patients were advised not to take analgesics until the last appointment and were monitored at each session while being encouraged to maintain treatment compliance.

### Therapeutic exercises

**Figure 1:**
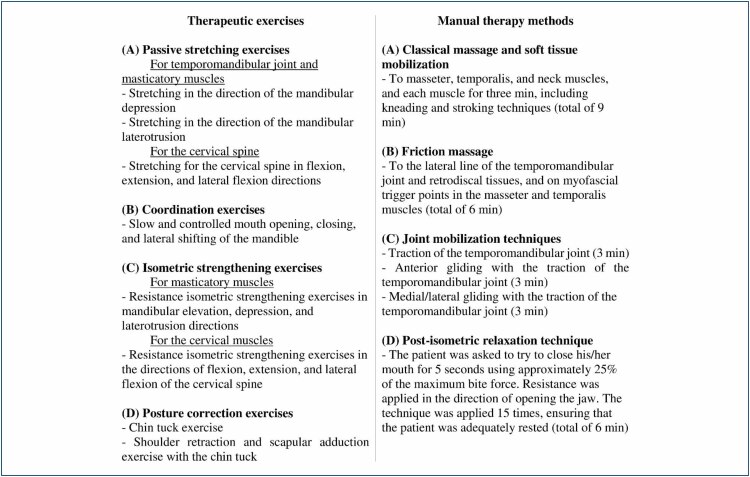
Detailed descriptions of therapeutic exercise programs and manual therapy methods.

Participants were instructed to perform stretching exercises to increase the range of motion by reducing pain and muscle spasm, coordination exercises for masticatory muscles, resistance isometric strengthening exercises, and posture correction exercises five times a day, two sets of 10 repetitions per set^5,15^. The exercise program is detailed in Figure 1.

### Transcutaneous electrical nerve stimulation

Conventional TENS for analgesia was applied to the painful areas. The intensity of the constant current (asymmetric biphasic conventional waves, frequency: 100 Hz, pulse width: 0.10 ms) was adjusted according to the patient's tolerance^7^.

### Manual therapy

MT techniques comprising classical massage and soft tissue mobilization to increase blood circulation and reduce tension and pain; friction massage to dissolve adhesions; and joint mobilization and post-isometric relaxation to release masticatory muscles, reduce pain, and increase flexibility and range of motion were applied^5,8,15^. The MT techniques are detailed in Figure 1.

### Clinical assessments

Age, body mass index (BMI), gender, and duration of the complaint were registered. Pain intensity, MMO, and MMA-DC of the masseter and TA muscles were assessed at baseline and after the 6-week treatment programs.

### Primary outcome

The pain intensity during jaw function was evaluated using a 10-cm visual analog scale (VAS). The patient was asked to mark pain intensity on a 10-cm line with "0" for "no pain" on the one end and "10" for the "most severe pain" on the other end, and the marked value was recorded in centimeters^3^.

### Secondary outcomes

To measure the pain-free MMO, the patient was asked to open their mouth wide until experiencing pain, and the vertical distance between the midpoints of the upper and lower incisors was measured with a ruler in millimeters^3^. The MMA-DC of the masseter and TA muscles on the affected side was evaluated by sEMG using a portable device (Delsys Trigno Wireless EMG System), with the same two protocols and methodology as in a previous study^3^. The first protocol was unilateral gum chewing, and the second was maximum voluntary clenching (MVC). The electromyography (EMG) signals from both protocols were analyzed using the same methodology as in the previous study^3^, and the percentage of MVC (%MVC) value, which is an expression of MMA-DC, was calculated for each masseter and TA muscles. After treatment, the increase in %MVC was accepted as an improvement in MMA-DC^3^.

### Sample size

The study used the G*Power program to calculate the sample size, based on a similar study by Tuncer et al.^15^, which considered a difference of 2.3 cm on the VAS as significant. With a power ratio of 90% and a type I error rate of 0.05, and considering a dropout rate of 15%, it was determined that 17 patients should be included in each group.

### Randomization

Patients were randomly divided into three groups of 17 patients each using the closed envelope method. The patients were instructed to randomly select a closed envelope to determine their group. In the allocation process, a 1:1:1 allocation was used with an equal distribution of participants between the groups through randomization.

### Statistical analysis

The study used IBM SPSS software version 24 for statistical analysis, using parametric tests and visual and analytical methods. Descriptive statistics were presented as mean±standard deviation or as numbers and frequencies. The chi-square test was used for gender distribution, and the one-way analysis of variance (ANOVA) test was used for evaluating continuous variables. The two-way mixed-design repeated-measures ANOVA was used to assess changes within groups over time and group-by-time interactions. The statistical significance level was accepted as p<0.05.

## RESULTS

A total of 51 of the 89 mTMD patients who met the inclusion criteria were randomly assigned to EG, TG, and MTG, with 17 patients in each group. The study was conducted with 100% compliance. The baseline demographic characteristics of the participants are given in Table 1. The groups were similar in terms of age, BMI, duration of complaint, and gender distribution (p>0.05).

**Table 1:** Baseline demographic characteristics of the groups.

Variables		Exercise group (n=17)		TENS group (n=17)		Manual treatment group (n=17)		F	p
		Mean±SD		Mean±SD		Mean±SD			
Age (years)		37.7±3.3		38.2±3.8		39.1±3.6		0.278	**0.68**
BMI (kg/m^2^)		25.5±1.6		24.6±1.6		25.6±1.7		0.638	**0.33**
Duration of complaint (years)		4.3±0.8		4.2±0.8		4.4±0.9		0.749	**0.38**
		n	(%)	n	(%)	n	(%)	χ^2^	p
Gender	Female	11	68.8	12	75.0	10	62.5	0.685	**0.59**
	**Male**	**5**	**31.2**	**4**	**25.0**	**6**	**37.5**		

TENS: transcutaneous electrical nerve stimulation; SD: standard deviation; BMI: body mass index; F: one-way analysis of variance test; χ^2^: chi-square test.

Table 2 shows the comparison of baseline and post-treatment scores for MMA-DC values, pain intensity, and MMO within and between groups. In all the three groups, decreases in pain intensity, increases in the MMA-DC values of masseter and TA muscles, and improvements in MMO were statistically significant after treatment (p<0.001). Considering group-by-time interactions, the decrease in pain intensity was statistically significantly greater in the MTG than in the TG, and the decrease in the TG was statistically significantly greater than that in the EG (p<0.001). The increase in MMO was the greatest in the MTG compared to the TG and EG (p<0.001), while the increases were similar in the TG and EG. After treatment, the improvements in %MVC values, expressing the MMA-DC of both masseter and TA muscles, were statistically significantly higher in MTG than in TG, and the improvements in TG were statistically significantly higher than those in EG (p<0.001) (Table 2).

**Table 2:** Comparison of baseline and post-treatment scores for masticatory muscle activity-during chewing values of the masseter and temporalis anterior muscles, pain intensity, and maximum mouth opening within and between groups.

Variables		Exercise group^x^ (n=17)	TENS group^y^ (n=17)	Manual treatment group^z^ (n=17)	Changes within the groups over time	Group-by-time interactions	Pairwise comparisons	η^2^
						F/p		
		Mean±SD	Mean±SD	Mean±SD	F/p			
Pain intensity (VAS, cm)	B	7.0±0.7	7.3±0.9	7.2±0.8	1,725.4/**p<0.001**	102.8/**p<0.001**	x–y, x–z, y–z	**0.82**
	AT	5.1±0.7	3.7±0.5	1.8±0.3				
MMO (mm)	B	19.1±3.6	18.7±3.3	18.4±3.2	1,067.2/**p<0.001**	116.7/**p<0.001**	x–z, y–z	**0.84**
	AT	24.8±4.5	27.5±4.9	36.9±5.8				
MMA-DC of the masseter (%MVC)	B	13.2±2.0	12.7±1.4	12.8±1.5	3,128.5/**p<0.001**	254.7/**p<0.001**	x–y, x–z, y–z	**0.91**
	AT	17.7±2.8	21.7±2.4	26.4±2.5				
MMA-DC of the temporalis anterior (%MVC)	B	11.2±1.5	11.1±1.4	11.6±1.9	2,191.7/**p<0.001**	218.3/**p<0.001**	x–y, x–z, y–z	**0.89**
	**AT**	**15.0**±**2.5**	**19.4**±**2.3**	**24.2**±**2.9**				

TENS: transcutaneous electrical nerve stimulation; SD: standard deviation; F: two-way mixed-design analysis of variance test; η^2^: effect size; VAS: visual analog scale; B: baseline; AT: after treatment; MMO: maximum mouth opening; MMA-DC: masticatory muscle activity-during chewing; %MVC: percentage of maximum voluntary contraction. Statistically significant values are denoted in bold. x: Exercise group; y: TENS group; z: Manual treatment group.

## DISCUSSION

To the authors’ best knowledge, the present study is the first to compare the effectiveness of therapeutic exercise, TENS, and MT on pain intensity, MMO, and MMA-DC in mTMD. This study showed that 6 weeks of therapeutic exercise was effective in improving the MMA-DC of masseter and TA muscles, pain intensity, and MMO in mTMD patients. The most effective approach for improving pain intensity, MMO, and MMA-DC was MT combined with exercise. TENS combined with exercise resulted in greater improvement in pain intensity and MMA-DC values compared to exercise alone.

The literature suggests approaches including exercise, TENS, and MT in the treatment of TMDs, especially to improve pain and MMO. However, it remains unclear which of these approaches, alone or combined, is more effective in treating pain and MMO in TMDs^8,9^. A recent systematic review stated that exercise is effective for TMDs, but the best results for pain and MMO can be achieved with a combination of exercise and MT^6^. Similarly, Tuncer et al.^15^ found that combining MT with exercise was more effective than exercise alone in improving pain and MMO in TMDs. The review study by Dorosz et al.^5^ suggested that MT was effective in improving pain intensity, MMO, and MMA in TMDs but also recommended that the effect of MT alone and in combination with other methods on these parameters should be investigated in further studies. The current systematic review, which reported that TENS can reduce pain and improve MMO and MMA in TMDs, also proposed comparing the effects of TENS alone and in combination with other treatments on these parameters^7^. The present study found that exercise significantly improved pain and MMO, with the most effective results occurring when exercise was combined with MT. Moreover, when exercise was combined with TENS, there was a greater decrease in pain intensity compared to exercise alone. Based on the results of the present study, in agreement with the literature, we recommend the combination of exercise and MT to further improve pain and MMO in mTMD.

TMD pain can interfere with MMA, leading to chewing difficulties and impairing essential jaw functions, thereby reducing the quality of life. It is therefore essential to focus on pain management to improve MMA in TMDs^3,5^. In the literature, clinical trials evaluating the effectiveness of conservative approaches on MMA with sEMG in TMD patients are quite limited. Most studies have reported only the immediate- or short-term effects of a single treatment modality on MMA during rest or at MVC^5,10-14^. Gavish et al.'s study on TMD patients found that an 8-week gum-chewing exercise reduced pain and significantly increased the masseter's MMA during MVC^10^. The authors highlighted that the increase in MMA may be related to the decrease in pain, but the effects of exercise on MMA should be investigated in further studies. The study by Ferreira et al.^11^ found that a 50-min TENS session significantly increased the MMA of the TA muscles during MVC and the MMA-DC values of the masseter and TA muscles. Another study showed that MT alone or combined with TENS similarly reduced pain in female TMD patients, and both approaches had similar effects on MMA at rest and during MVC^14^. Barone et al.^13^ stated that single-session 1-min rhythmic joint mobilization to the TMJ improved pain intensity and MMO in TMD patients but did not significantly alter masticatory muscle electromyographic activity. Manzotti et al.^12^ declared that a 30-min single-session osteopathic MT improved MMA of the masticatory muscles, but further long-term clinical studies are needed to confirm these findings. The current study revealed significant increases in MMA-DC values in all the three groups, with MTG showing the greatest increase, followed by TG and EG. We also observed that the decrease in pain intensity was the greatest in MTG, followed by TG and EG. Given the knowledge that pain may be associated with MMA in TMDs^3,5,10^ and the findings of our study, it can be concluded that a greater decrease in pain with combined treatments may lead to a further improvement in MMA-DC. For mTMD, we propose a combination of exercise and MT or, if this is not possible, a combination of exercise and TENS, instead of exercise alone, to achieve a greater improvement in pain intensity and MMA-DC.

The study has some limitations. First, given the suggestion that conservative treatments may be more effective on the MMA of masticatory muscles in mTMD^5^, only patients diagnosed with mTMD were included, and other TMD subdiagnostic groups, such as disc displacement and arthralgia, were not included. Second, the study data were obtained by assessing participants before and after the 6-week treatment programs. However, the treatment groups were not followed up for 6 months or 1 year to assess the long-term effects of the treatments. Further studies, including different TMD subdiagnosis groups and evaluating the long-term effects of these treatments, are recommended. On the other hand, the strength of the present study is as follows: to our knowledge, this is the unique randomized controlled trial comparing the effectiveness of exercise, TENS, and MT on the MMA-DC of the masseter and TA muscles in mTMD, and is expected to contribute significantly to the literature.

## CONCLUSION

Exercise is an effective method for improving pain intensity, MMO, and MMA-DC in mTMD. A combination of exercise and MT may provide the greatest therapeutic effect on these parameters. Additionally, the combination of exercise with TENS may further improve pain and MMA-DC compared to exercise alone. For mTMD, we recommend a combination of exercise and MT or, if this is not possible, a combination of exercise and TENS, instead of exercise alone, to achieve a greater improvement in pain intensity and MMA-DC.

## Data Availability

The datasets generated and/or analyzed during the current study are available from the corresponding author upon reasonable request.
